# Carbon Monoxide Therapy: Evidence and Prospects for Preventing and Treating Retinal Diseases

**DOI:** 10.3390/biom16020291

**Published:** 2026-02-12

**Authors:** Mathew Reese Land, Marybeth Koepsell, Noah Nussbaum, Edward Gomperts, Andrew Gomperts, Menaka C. Thounaojam, Ravirajsinh N. Jadeja, Pamela M. Martin

**Affiliations:** 1 Department of Biochemistry and Molecular Biology, Medical College of Georgia, Augusta University, Augusta, GA 30912, USA; reese.land@cahabamedicalcare.com (M.R.L.); mkoepsell@augusta.edu (M.K.); 2 Department of Music, Furman University, Greenville, SC 29613, USA; nussbaumnoah@gmail.com; 3 Hillhurst Biopharmaceuticals, Inc., Montrose, CA 91020, USA; edwardgomperts@gmail.com (E.G.); agomperts@hillhurstbio.com (A.G.); 4 Department of Biomedical Sciences, School of Graduate Studies, Meharry Medical College, 1005 D.B. Todd Jr. Blvd, Nashville, TN 37208, USA; menaka.thounaojam@mmc.edu; 5 Meharry-Vanderbilt Alliance, Meharry Medical College, Nashville, TN 37208, USA

**Keywords:** heme oxygenase-1, ischemic retinopathy, carbon monoxide, retinal diseases

## Abstract

In carbon monoxide (CO) therapy, CO is administered at low concentrations as a controlled solution; this approach enables the drug to achieve its cytoprotective properties, including anti-inflammatory, anti-apoptotic, and vasodilatory effects. CO therapy, initially reported to benefit cardiovascular and pulmonary conditions, is now used to treat ocular diseases in preclinical models. Carbon monoxide, a compound most famously known for its deleterious effects, is receiving more attention as a potential therapeutic candidate in ocular medicine. In a few studies, controlled low-dose CO therapy has shown anti-inflammatory and anti-apoptotic effects in various models of retinal disease (such as retinal ischemia-reperfusion injury, optic nerve crush, ocular hypertension, and autoimmune uveitis). We have summarized the clinical and preclinical findings, along with the potential therapeutic value of CO, in this review. In this context, the current and emerging CO delivery methods are also described, with a focus on exploring their safety, efficacy, and applicability in retinal disorders. Although a strong preclinical paradigm exists, clinical translation is limited at best. While some trials indicate acceptable safety levels for inhaled CO or CORM-based interventions, these results have not been robust or reproducible. Bridging this efficacy gap will rely on enhanced delivery strategies, stringent PK/PD-informed dosing, and mechanism-specific endpoint-based trials.

## 1. Introduction

Degenerative retinal diseases precipitated by diabetes (diabetic retinopathy), premature birth (retinopathy of prematurity), increased intraocular pressure (glaucoma), and aging (age-related macular degeneration) represent leading causes of visual loss and blindness worldwide [1,2]. Despite significant advances in understanding the molecular mechanisms underlying these conditions, effective early interventions remain limited. Current therapies—including anti-VEGF agents, corticosteroids, laser photocoagulation, and surgical procedures—have improved outcomes for many patients but are often associated with limitations such as incomplete efficacy, recurrence of disease, adverse effects, and the need for repeated invasive treatments [3,4,5].

For example, anti-VEGF therapy has revolutionized the management of neovascular retinal diseases but requires frequent intravitreal injections and may not be effective in all patients [3,4,5]. Corticosteroids, while potent anti-inflammatory agents, carry risks of cataract formation and elevated intraocular pressure. Laser therapy can cause collateral damage to healthy retinal tissue, and surgical interventions are typically reserved for advanced stages of disease [3,4,5].

Moreover, the posterior segment of the eye remains a challenging target for drug delivery due to anatomical and physiological barriers [6,7,8]. Non-invasive yet effective delivery systems are still under development, and many promising compounds fail to reach therapeutic concentrations in retinal tissues when administered systemically or topically [6,7,8].

Given these limitations, there is a pressing need for novel therapeutic strategies that are both effective and minimally invasive. One such emerging approach is the use of carbon monoxide (CO) therapy. Traditionally viewed as a toxic gas, CO has demonstrated cytoprotective properties at low concentrations, including anti-inflammatory, anti-apoptotic, and vasodilatory effects. This review explores the therapeutic potential of CO and heme oxygenase-1 (HO-1) induction in ocular diseases, summarizing current evidence and discussing delivery strategies, safety considerations, and future directions.

CO is classically considered a toxic gas. This is related to its well-known property of binding with high affinity to hemoglobin to generate carboxyhemoglobin (COHb). COHb decreases hemoglobin’s ability to carry oxygen, contributing to hypoxemia, tissue hypoxia, and, if in high concentrations not addressed rapidly, death. CO is also generated physiologically in the body as a byproduct of heme degradation in a reaction catalyzed by heme oxygenase enzymes. Heme oxygenase 1 (HO-1) is essential to endogenous CO production. Several reports have highlighted the benefits of enhanced HO-1 expression and activity in a variety of disease model systems including in animal models of intestinal [9,10,11], hepatic [12,13,14], lung [15,16,17], renal [18,19], cardiac [20,21,22], and ocular ischemic reperfusion injury [23,24,25]. However, the detailed mechanisms to explain these effects, particularly whether or how they relate directly or indirectly to the modulation of CO levels, are unclear.

An increasing number of studies expose the robust and direct benefits of CO in a wide variety of clinical and pre-clinical scenarios, sparking increased interest in CO therapy. This fascinating area of research showcases CO in a light that contrasts starkly with traditional views. The current review focuses on the potential therapeutic effects of CO that could be realized in the eye, providing information on the mechanism(s) of action, available pre-clinical and clinical evidence relevant to the utility of CO in retinal diseases, delivery methods, and safety considerations, as well as prospects and future directions.

Despite extensive preclinical support, translation of CO-based therapeutics to human disease has been challenging. Most early-phase clinical studies have established feasibility and short-term safety but have not demonstrated durable or clinically meaningful efficacy across target indications. This divergence between preclinical promise and clinical outcomes underscores a critical efficacy gap and motivates a more analytic approach to dosing, delivery, and pharmacology, as well as the selection of indications and endpoints that are mechanistically congruent with CO’s modes of action.

## 2. Literature Search and Selection Criteria

A systematic literature search involving PubMed, Scopus, and Google Scholar was performed to identify peer-reviewed and preprint studies regarding the biological significance of carbon monoxide (CO) and its therapeutic action in ocular diseases. The most current search was conducted on 20 November 2026. The search terms used were: “carbon monoxide,” “CO therapy,” “heme oxygenase-1,” “HO-1,” “cytoprotection,” “retinal disease,” “ischemic retinopathy,” “retinal ischemia-reperfusion,” “uveitis,” and “ocular hypertension.” Inclusion criteria included studies that had evaluated CO signaling, CO-releasing molecules (CORMs), heme oxygenase pathways, or that assessed the therapeutic utility of CO from the perspective of ocular or retinal models, including in vitro, in vivo, and clinical studies. We limited our search of ClinicalTrials.gov, for the following: “carbon monoxide,” “CO therapy,” “CORM,” and included interventions using systemic or localized CO delivery, CO-releasing molecules, and inhaled CO. All articles were excluded for lack of English translation, not available full text references, or for not addressing mechanisms of CO-mediated treatment of ocular pathology. Screening of reference lists of included studies was also performed to identify further relevant literature.

## 3. Mechanisms of Action for Low-Dose CO

CO is produced during the enzymatic degradation of heme by heme oxygenase-1 (HO-1), a stress-inducible enzyme that responds to various physiological insults. These effects are tightly regulated and dose-dependent, with low-dose CO triggering beneficial responses without inducing toxicity.

**Figure 1:**
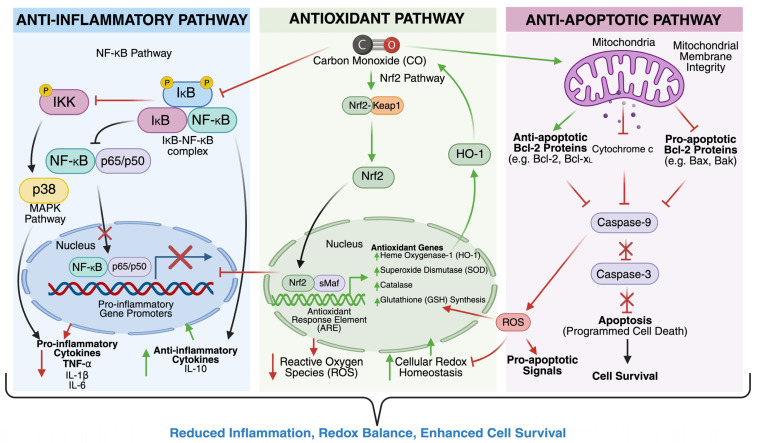
Carbon Monoxide–Heme Oxygenase Molecular Pathway. Activation of the PI3K (phosphoinositide 3-kinase)/Akt (protein kinase B)/mTORC1 (mechanistic target of rapamycin complex 1) pathway leads to nuclear translocation of Nrf2 (nuclear factor erythroid 2–related factor 2), which binds to SRE (stress response elements) and induces expression of HO-1 (heme oxygenase 1). HO-1 catalyzes the degradation of heme into ferrous iron (Fe^2+^), biliverdin (converted to bilirubin via biliverdin reductase), and carbon monoxide (CO). CO exerts anti-apoptotic effects through activation of sGC (soluble guanylate cyclase) and CREB (cAMP response element-binding protein), modulating pro- and anti-apoptotic proteins Bax (B-cell lymphoma-2-associated X protein) and Bcl-2 (B-cell lymphoma-2). Additionally, microglial activation and cytokine signaling (IL-6, interleukin-6; IL-17, interleukin-17; IL-10, interleukin-10) influence inflammatory responses via AIF-1 (allograft inflammatory factor 1), TNF-α (tumor necrosis factor alpha), and NF-κB (nuclear factor kappa-light-chain-enhancer of activated B cells) pathways. The pathway integrates oxidative stress regulation, apoptosis control, and neuroinflammation, highlighting the cytoprotective role of HO-1 and CO. The illustration was created in BioRender.

The cytoprotective actions of CO begin with the induction of HO-1, which catalyzes the breakdown of heme into biliverdin, free iron (Fe^2+^), and CO. Biliverdin is subsequently converted to bilirubin by biliverdin reductase, and both products contribute to the cellular antioxidant defense system [26] (Figure 1). HO-1 expression is regulated by the transcription factor Nrf2, which is activated via the PI3K/Akt/mTORC1 signaling pathway. Upon activation, Nrf2 translocates to the nucleus and binds to antioxidant response elements (AREs), promoting the transcription of HMOX1 and other genes involved in cytoprotection [27,28]. This pathway has been extensively studied in models of ischemia-reperfusion injury, neurodegeneration, and inflammation, underscoring its central role in CO-mediated cellular resilience.

CO also plays a critical role in modulating inflammation. It suppresses the activation of immune cells such as microglia and macrophages, leading to a reduction in pro-inflammatory cytokines, including TNF-α, IL-1β, and IL-6 [29,30,31]. Simultaneously, CO enhances the production of anti-inflammatory cytokines like IL-10, promoting the resolution of inflammation [32]. In the central nervous system, CO downregulates allograft inflammatory factor-1 (AIF-1), a marker of microglial activation, thereby mitigating neuroinflammatory responses [33]. These effects are mediated through mitogen-activated protein kinase (MAPK) signaling cascades, particularly p38 MAPK and ERK1/2, which regulate cytokine transcription and immune cell behavior (Figure 1). Studies using inhaled-CO and CO-releasing molecules (CORMs) have confirmed these anti-inflammatory effects in various disease models, including sepsis, lung injury, and transplant rejection [34].

In addition to its immunomodulatory properties, CO enhances antioxidant defenses and maintains redox homeostasis [35]. Through HO-1 induction, CO increases intracellular levels of bilirubin, a potent scavenger of reactive oxygen species (ROS). It also modulates the activity of key antioxidant enzymes such as superoxide dismutase (SOD), catalase, and glutathione peroxidase, thereby reducing oxidative damage [35,36]. CO has been shown to inhibit NADPH oxidase, a major source of superoxide in inflammatory cells, further limiting ROS production. Moreover, CO may interact with reactive nitrogen species (RNS) to form carbonate radicals, which modulate redox-sensitive signaling pathways and prevent excessive oxidative injury [35]. These mechanisms have been validated in cardiovascular, renal, and neuroprotective models, demonstrating the role of CO in preserving cellular integrity under stress.

CO’s anti-apoptotic effects are equally significant. It activates soluble guanylate cyclase (sGC), leading to increased cyclic GMP (cGMP) levels and subsequent activation of cAMP response element-binding protein (CREB), a transcription factor that promotes cell survival [37] (Figure 1). CO also influences the expression of Bcl-2 family proteins, inhibiting pro-apoptotic Bax and enhancing anti-apoptotic Bcl-2, thereby stabilizing mitochondrial membranes and preventing cytochrome c release [38,39]. Furthermore, CO inhibits the activation of caspase-3 and caspase-9, key mediators of the intrinsic apoptotic pathway [39,40]. These protective effects have been observed in models of ischemia, neurodegeneration, and organ transplantation, where CO treatment significantly reduced tissue damage and improved functional outcomes.

## 4. Mechanistic Insights and Controversies from Preclinical Models

The benefits of CO therapy have also been realized in the eye, where the impact of CO therapy has mainly been cytoprotective. Indeed, many ocular disease models have been used to study the effects of CO. The most common model is the rodent model of ocular ischemia-reperfusion injury (IRI). IRI occurs when the blood supply to a tissue, such as the retina, is inadequate; thus, the metabolic and oxygen needs of the tissue are not met [41,42]. Such ischemia can lead to angiogenesis. In the retina, the new vessels that form in response to tissue ischemia are fragile and prone to hemorrhage in the vitreous. The reperfusion of previously under-perfused tissue areas can additionally yield further damage due to the generation of reactive oxygen species (ROS) and pro-inflammatory molecules [43,44,45]. Further, if left untreated, these new vascular tufts can lead to tractional retinal detachment and vision loss [41,42,46]. Tissue ischemia occurs commonly in eye diseases such as glaucoma, diabetic retinopathy, retinopathy of prematurity, and retinal vasculature occlusions [42,47,48]; thus, information gleaned from the ocular IRI model is highly relevant

The efficacy of CO therapy has also been evaluated in models of optic nerve crush (ONC) [49,50] and ocular hypertension [51,52], models that replicate key processes in human glaucoma and traumatic optic neuropathy, and in the rat model of autoimmune uveitis, a model consistent with robust general inflammation in the posterior segment [53]. In these models, CO was found to reduce intraocular pressure and related ocular hypertension, and limit inflammation through the reduction in interleukin-17 (IL-17) expression and increased expression of the anti-inflammatory cytokine IL-10, resulting in improved histological scores.

Ulbrich et al., using a rat model of retinal IRI, found that exogenously delivered CO reduced expression of the pro-inflammatory cytokine interleukin-6 (IL-6); this effect of CO, mediated by soluble guanylate cyclase (sGC), played a key role in protecting retinal ganglion cells (RGCs) from IRI-induced cell death [54,55]. Postconditioning with inhaled CO (250 ppm) in a rat IRI model reduced activation of microglia-neuroglia that can secrete pro-inflammatory cytokines upon activation. Moreover, retinal thickness was preserved in the CO-treated group compared to the IRI + air group [56]. Additional research showed that CO, given post-IRI in a rat retina model, reduces allograft inflammatory factor 1 (AIF-1) expression, a protein implicated in microglial activation; thus, inflammation was reduced while RGCs were protected [57]. Schallner et al. found that postconditioning with 250 ppm inhaled CO reduced NF-kB expression and DNA binding in their rat model of retinal IRI; thus, inflammation and RGC death were reduced [58]. Preconditioning with 250 ppm inhaled CO in a rat model of retinal IRI has been shown to increase heat shock protein 70 (HSP70) expression, a cytoprotective chaperone [57]. 

Table 1 highlights the findings of these and other preclinical studies on the use of CO delivery to improve various ocular disease conditions.

CO, like HO-1, has extensive anti-apoptotic effects. Much evidence reveals that CO protects RGCs from apoptosis via suppression of caspase-3 and caspase-9 [49,50,56,57,58,59]. The use of CORMs [59] in combination with postconditioning with 250 ppm of inhaled CO [58] reduced expression of Bax and increased expression of Bcl-2 in rat models of retinal IRI. Moreover, preconditioning with inhaled CO (250 ppm) in a rat retinal IRI model increased the activity of cAMP response element-binding protein (CREB), a transcription factor implicated in neuronal plasticity, growth, and survival [57]. The effects of CO on MAPKs have been controversial. For example, evidence suggests that CO stimulates p38 MAPK expression in models of retinal IRI to exert anti-apoptotic effects [56,57,59]. In contrast, Schallner et al. noted less apoptosis secondary to decreased phosphorylation of p38 MAPK with inhaled CO (250 ppm) given post-IRI in a rat model [58]. Several researchers noted that the timing of CO administration could differentially affect the expression of p38 MAPK [57,58]. In studies that showed increased p38MAPK expression, CO treatment, whether via CORM or inhalation, was preconditioned or administered quickly (<3 h) [56,59] after IRI. However, administration of 250 ppm inhaled CO immediately after, 1.5 h, or 3 h post-IRI resulted in decreased p38 MAPK activation [58]. Research on CO’s effect on ERK1/2 MAPK has proven contradictory as well. In their rat model of IRI, Schallner et al. noted increased ERK1/2 phosphorylation after postconditioning with 250 ppm of inhaled CO; however, inhibition of ERK1/2 did not abrogate CO’s protective effects on RGCs [58]. Ulbrich et al., using CORM ALF-186 in a rat model of retinal IRI, found that CO administration reduced ERK1/2 phosphorylation [59]. The apparent differences in CO and CO-induced MAPK responses across studies seem to reflect the context-dependent nature of MAPK signaling, which differs according to cell type, post-injury timing, and experimental model; therefore, these data should be considered indicative of model-specific adaptations and not mechanistic discrepancies.

The aforementioned studies demonstrate the controversy that surrounds CO’s actions on MAPK signaling pathways. Such discrepancies highlight the need for future studies on the effects of CO on MAPKs. Although research on CO’s direct effects on HO-1 in an ocular context is lacking, accumulating evidence suggests that exogenously delivered CO can, in turn, increase HO-1 expression. Yang et al., in their study of STAT3 activation in bovine endothelial cells, discovered that CORM-derived CO increased HO-1 protein levels [60]. Moreover, CO, whether inhaled or from CORMs, increases Nrf2 mRNA and subsequently HO-1 mRNA expression and enzyme activity [61]. Further study in a mouse model of cerebral ischemia revealed that inhaled CO (post-conditioned, 250 ppm) aids Nrf2 translocation into the nucleus, resulting in increased HO-1 expression [62]. Additional evidence from a study of CORM-treated rat pheochromocytoma cells suggests that CO increases phosphorylation of Akt; inhibition of PI3K decreased cell viability [63]. Taken together, CO seems to induce HO-1 expression via a PI3K/Akt/Nrf2 pathway similar to that shown previously in Figure 1 [61,62,63]. Zuckerbraun et al., using a rat model of liver injury, suggested that CO stimulates iNOS, producing nitric oxide (NO) that then stimulates HO-1 [64]. In contrast, Schallner et al. did note that inhaled CO treatment post-IRI in the rat retina resulted in decreased HO-1 mRNA and protein expression. However, they state that this is likely due to a CO-mediated decrease in oxidative stress; since oxidative stress can stimulate HO-1, removing it would naturally decrease HO-1 expression [58]. In any case, further studies on how CO interacts with HO-1 in the eye are needed. CO is known to activate redox-sensitive transcriptional factors and signaling that result in the induction of HO-1 expression. However, exogenous CO can reduce oxidative stress and, hence, also reduce the upstream signals for HO-1 transcription, resulting in decreased HO-1 expression. This reflects reduced oxidative stress rather than the suppression of the pathway per se. This bidirectionality is likely influenced by baseline oxidative stress, timing and duration of CO, tissue compartment, and the specific readout (mRNA, protein, or enzymatic activity).

In a blue light-induced injury model, CORM-2 and CORM-3 demonstrated cytoprotective and anti-inflammatory effects in ARPE-19 cells via NF-kB activation. Compared to CORM-3, CORM-2 was more efficient in increasing levels of GSH synthesis enzymes, resulting in stronger cytoprotective effects in RPE cells. Lastly, this study demonstrated that CORM-2 and CORM-3 inhibit migration in VEGF-induced endothelial cells [65]. 

**Figure 2:**
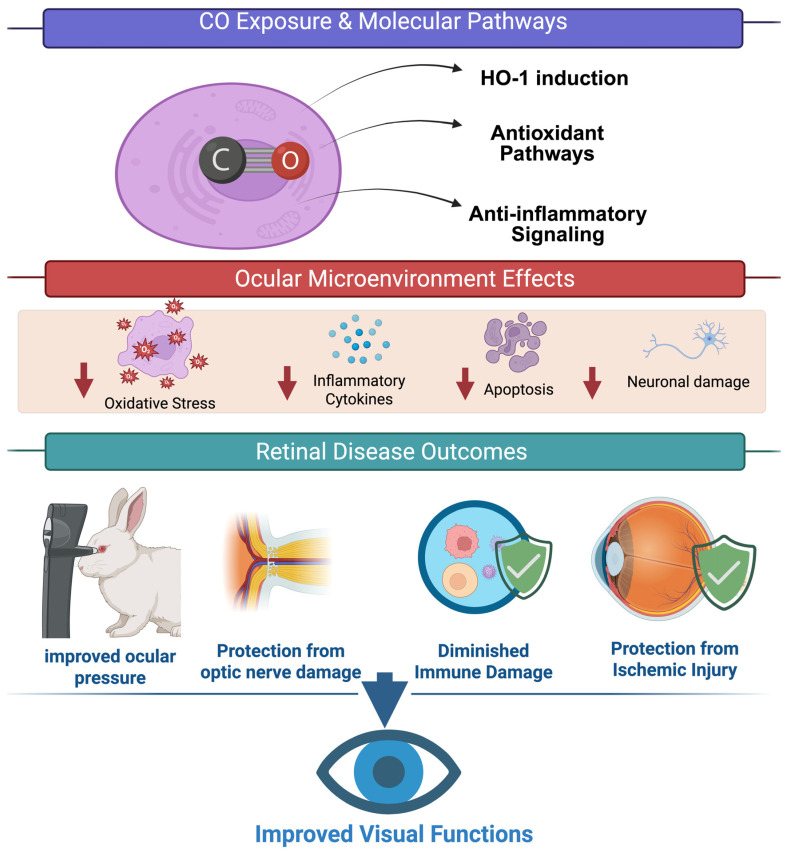
Overview of CO-mediated signaling pathways in retinal protection. Carbon monoxide (CO) provides protection against retinal insults by activating protective pathways, such as hemeoxygensase-1, antioxidant, and anti-inflammatory responses. Activation of these pathways reduces oxidative stress, inflammation, and cell injury, thereby improving retinal outcomes and ultimately preserving vision. The illustration was created in BioRender.

As summarized in Figure 2, the mechanistic pathways described in this section converge on key modifications in the ocular microenvironment, which in turn underlie the therapeutic effects observed in preclinical models.

## 5. Clinical Evidence

Most clinical trials studying the therapeutic efficacy and safety of carbon monoxide are focused on treating respiratory disease using inhaled CO (Table 2). As of right now, there is no therapy for the inflammation that occurs from smoking-related chronic obstructive pulmonary disease (COPD). Researchers found that low-dose CO inhalation in patients with stable COPD was feasible and safe relative to the peak carboxyhemoglobin levels of 3.1–4.5%. CO inhalation also led to reduced sputum eosinophils and improved responsiveness to methacholine in patients with stable COPD. Their findings indicate that CO inhalation might have therapeutic effects in COPD [66].

Several studies have looked at CO inhalation efficacy in treating acute respiratory distress syndrome (ARDS), a prevalent disease for which there is no effective pharmacologic therapy. One study administered bronchoscopic instillation of endotoxin (LPS) in healthy volunteers to model a pulmonary inflammatory response. Although CO did not demonstrate significant anti-inflammatory effects in the pilot study, data analysis for the main study remains ongoing [NCT00094406]. Another study assessed the efficacy of inhaled CO to treat idiopathic pulmonary fibrosis (IPF) in an ambulatory setting. Despite increases in COHb, CO treatment did not significantly raise matrix metalloproteinase-7 (MMP7), a biomarker in IPF. This study demonstrated that inhaled CO is well tolerated and can be safely administered in patients with IPF. However, there were no observed differences in physiologic measures, acute exacerbations, hospitalization, death, or patient-reported outcomes between the subjects treated with CO and the control group [66].

One study conducted a dose escalation trial to determine the feasibility and safety of inhaled CO in patients with sepsis-induced ARDS. By utilizing a personalized inhaled CO dosing algorithm, researchers found that the Coburn-Forster-Kane (CFK) equation was highly accurate at predicting COHb levels, which would ensure COHb levels remain in the target range during future clinical trials [67]. To further this research, an ongoing study is exploring the safety and accuracy of the same CFK equation algorithm to achieve COHb of 6–8% in mechanically ventilated patients with sepsis-induced ARDS [NCT04870125]. This clinical trial has progressed to Phase II to assess the efficacy of low-dose inhaled CO in mechanically ventilated patients with ARDS [NCT03799874].

The negative or inconclusive efficacy seen across inhaled CO trials for COPD, ARDS, and IPF likely reflects several translational barriers rather than an absence of biological activity. First, the strict safety-driven COHb limits used in human studies constrain dosing to levels that may modulate inflammatory biomarkers but remain insufficient for sustained therapeutic engagement of CO-responsive pathways. Second, the PK/PD profile of inhaled CO, characterized by brief systemic exposure and rapid hemoglobin binding, may fail to reproduce the continuous or tissue-localized signaling effects observed in preclinical models. Finally, the complexity and heterogeneity of human lung diseases, including advanced structural damage, comorbidities, and ongoing environmental or infectious insults, can overwhelm modest pathway-specific interventions. Together, these factors help explain why promising preclinical findings have not yet translated into clear clinical efficacy.

An oral liquid CO drug product, HBI-002, is also being studied in the clinic. A Phase 1 clinical trial investigating HBI-002 in healthy adult subjects has been completed [NCT03926819]. In this open-label study, HBI-002 was administered to a total of 20 subjects in a single ascending dose phase followed by a multiple daily dose phase with daily dosing for 7 days. This study demonstrated appropriate safety, with no Serious Adverse Events and only Grade 1 Adverse Events, as well as dose-dependent pharmacokinetics. In addition, a Phase 2a clinical study of HBI-002 in subjects with SCD is ongoing [NCT06144749]. This study is an open-label, ascending multiple-dose study with once daily dosing for 14 days, assessing safety, pharmacokinetics, and proof-of-concept efficacy.

To establish greater transparency concerning the strength and maturity of evidence showing the therapeutic advantage of carbon monoxide (CO) in ocular diseases, we clearly differentiate between cellular, animal, and human data across sections of the manuscript. When moving between levels of evidence, we distinguish between and compare the relative strengths, generalizability, and limitations of different stages—from mechanistic in vitro studies, to in vivo efficacy in animal models, and ultimately to early-phase clinical studies primarily concerned with tolerability. Structuring this evaluation allows for a granular evaluation of how well preclinical results translate to human disease and makes clear just how much of what has been described is the best available evidence for the ocular indications mentioned to date. More critically, although the available clinical evidence indicates the potential feasibility and short-term safety of controlled, low-dose CO inhalation, we do not have robust or reproducible evidence of effectiveness. Despite a variety of clinical trials reporting the presence of benefits that can be varied and often fail to improve clinically. Therefore, no confirmed clinical effectiveness against inhaled CO or CO-releasing molecules has been demonstrated so far. We explicitly identify this ‘efficacy gap’ and treat it as a key limitation to our field rather than as a secondary observation. To further refine the calibration of assertions, we supply a brief evidence map (Table 3) detailing (for each ocular indication) the specific experimental model or type of data, reported biological or clinical effects, the presence or absence of human evidence, and key limitations. In light of these findings, they collectively indicate that future clinical trials should now be structured in relation to PK/PD-informed dosing, mechanistically consistent endpoints, and appropriate patient populations, as opposed to general syndromic outcomes.

## 6. Delivery Methods

### 6.1. Inhalation Therapy

Of course, the worry with inhaled CO is the risk of CO poisoning. This is due to the displacement of oxygen (O_2_) from hemoglobin (Hb) by CO, which has a much higher affinity for Hb than O_2_ [68,69,70]. In addition, CO bound to Hb increases Hb’s affinity for O_2_, thus inhibiting O_2_ release to the tissues [69,70,71]. These effects combine to result in tissue hypoxia [70,71]. A carboxyhemoglobin (COHb; fraction of hemoglobin bound to CO) level of 15 to 20% does not seem to have severe side effects; thus, this is presumed to be the threshold for human CO tolerance [72]. More than ten human studies with inhaled CO have shown no adverse effects [73]. Resch et al. found that inhaled CO increased retinal and choroidal blood flow in human volunteers after inhalation of 500 ppm CO for 1 h. The maximum COHb (Dose units (ppm for inhaled gas, mg·kg^−1^ for systemic formulations) are routinely converted to COHb%, the standard readout of systemic CO exposure (Dose unit ppm for inhaled gas is routinely converted to COHb%, the standard readout of systemic CO exposure) level was 8.7%, but CO inhalation was well-tolerated, and no headaches or discomfort were reported [74]. Inhaled CO (100 or 150 ppm) in a clinical trial with COPD patients was well-tolerated. Median COHb was less than 4% with both CO doses, [75]. Ren et al. maintained 10% COHb via inhaled CO (0.4% CO in air) for 8 h in human volunteers without adverse events [76]. Inhaled CO, either 250 ppm for 2 h or 500 ppm for 1 h, was used in a human model of LPS-induced systemic inflammation; COHb levels did not exceed 8%, vital signs remained normal, and no adverse events were noted [77]. In a recent study of interstitial pulmonary fibrosis patients, inhaled CO (100 to 200 ppm) produced maximum COHb levels of 2.24 to 3.82%. The CO treatment was well-tolerated, and no statistical difference in adverse events was noted between the control and CO-treated groups [67]. Based on the above results, CO treatment to achieve low COHb levels is safe and well-tolerated by human subjects. However, human studies with inhaled CO typically fail to produce therapeutic results. Despite CO’s safety in human models, inhaled CO in human trials of COPD [75], LPS-induced systemic inflammation [77], and interstitial pulmonary fibrosis [67] have failed to produce efficacious results. This could be due to underdosing. However, inhaled CO suffers from several disadvantages. Patient compliance is a substantial barrier for inhaled CO. Although inhaled drugs are accepted intraoperatively, there is substantial patient and healthcare provider resistance to inhaled drugs in a nonoperative setting. Dosing accuracy is another substantial issue. Although intraoperative dosing of gases can be well controlled due to the constant monitoring of blood gases and other measures, with nonoperative therapeutic gas dosing there is a substantial risk of a patient not breathing the correct dose, whether due to incorrectly used administration equipment (e.g., incorrectly applied breathing mask), variable inhalation (differences in breathing volume and rate during administration), poor compliance with duration of inhalation, or changes in lung function (e.g., decreased due to infection). Also, inhaled CO lacks specificity, as it is systemically distributed once it reaches the lungs; thus, it is difficult to control its absorption, distribution, and tissue targeting [72,78,79]. Moreover, Hb can serve as a “trap” for inhaled CO. That is, when the CO is bound to Hb, it may be restricted from reaching the site of injury in target tissues [80]. Additionally, CO leaks can be a risk to clinical staff and patients [78].

### 6.2. Carbon Monoxide-Releasing Molecules (CORMs)

The use of CORMs as a therapeutic has been a fascinating area of research. Indeed, this treatment modality holds the potential for the use of CO as therapy without the downsides of inhaled CO. CORMs are molecules composed of a backbone that release CO under certain conditions and/or stimuli [80]. CORMs are also known as CO prodrugs. The backbone moiety of CORMs can consist of a range of molecules, including organometallic and organic molecules, and both large and small molecules. CORMs have been designed to be dosed orally, intravenously, and through other routes of administration. In fact, CORMS have been designed such that oxidative environments, light, heat, pH changes, etc., can be used to liberate CO from these CORMs, making them attractive candidates for treatment in a variety of ocular diseases [80]. An additional advantage of certain, non-oral CORMs that are tissue-targeted over inhaled CO is minimal COHb elevation with treatment due to the tissue specificity of these CORMs [72,80]. Also, since these targeted CORMs theoretically only release CO when the target tissue is reached, the liberated CO does not fall victim to the “Hb trap” [72]. Perhaps the most attractive aspect of CORMs is the customization offered by the ancillary ligands attached to them; these can be used to “fine-tune” the stability, solubility, pharmacokinetics, and CO-releasing trigger of the CORM [79,80]. Indeed, certain CORMs have been used in animal models of ocular disease with positive results. For example, CORM-A1 had anti-inflammatory effects in a rat model of autoimmune uveitis [53], while ALF-186 has been shown to reduce inflammation and apoptosis [54,59] in rat models of IRI. However, CORMs have their downsides as well. A key concern is the pharmacological, metabolic, and toxic characteristics of the CORM backbone after CO release [72,79,80]. Organometallic CORMs are often made using a variety of transition metals, such as Cr, Mo, Mn, Re, Fe, and Ru [80], all of which can present toxicology concerns. For example, ruthenium-based CORMs (e.g., CORM-2) have been shown to be cytotoxic, and evidence suggests that Mn is neurotoxic, so the fate of the CORM backbone warrants concern [81]. At least in part due to these toxicity concerns, small molecule organometallic CORMs have thus far not been studied in the clinic.

The CORMs that have advanced farthest in development are large molecule organometallic CORMs, the PEGylated COHb drugs. These intravenous drugs typically consist of human or bovine cell-free COHb molecules to which polyethylene glycol (PEG) has been attached. Ten clinical trials have been completed with this class of CORM, including a total of 320 study subjects in both Phase 1 and 2 studies. Studies with these molecules, which have included a total of 320 subjects [73], have reported a toxicology profile similar to that reported for other cell-free hemoglobin (Hb) products (also termed Hb-based oxygen carriers). Reported AEs include cardiovascular issues (hypertension, troponin I increase, and a few myocardial infarction occurrences) as well as hematuria. These AEs are attributed to the effects of circulating free Hb, heme, and ferric iron and/or scavenging of nitric oxide (NO) by the heme moiety [82,83]. The companies developing PEG-COHb drug products focused on acute use of these molecules, rather than chronic use, likely due to the impact of free Hb toxicity. At present, it does not appear that any of these molecules are advancing in development.

There is increasing interest in developing “CO in a pill”, a solid oral CO drug that enables local release in the GI system. The potential to dose orally would provide a substantial advantage over inhaled CO and non-oral CORMs as oral dosing is the preferred modality for patients, providing improved ease of administration and patient compliance. However, as with inhaled CO, oral CORMs present with the downside of systemic exposure. Although oral organometallic CORMs have been developed (e.g., CORM-401) [84], to avoid the abovementioned toxicity of organometallic backbones, organic CORMs (also termed organic CO prodrugs) are also being developed, and drug product candidates have been developed with the goal of minimizing the absorption of the backbone after CO release [85,86]. A number of organic CORMs are in early stage development, for example oxalyl saccharin, which has been studied in initial pharmacokinetic studies in mice, and none so far has advanced to the clinic [87].

The advent of nanomaterials offers another solution to potential organometallic CORM backbone toxicity [88]. These materials, which can be conjugated to the organometallic CORM, may reduce the metal toxicity of the backbone. In addition, nanomaterials can be used to increase the solubility, stability, CO payload, and specificity of organometallic CORMs [88]. Thus, nanomaterials could enhance the therapeutic effects of organometallic CORMs while reducing any potential side effects.

### 6.3. Oral Liquids Containing CO

Another method of CO administration that holds great promise is that of liquid CO formulations. These CO therapeutics contain CO that is not chemically bound to any molecule, and are designed to circumvent the aforementioned cons of inhaled CO and CORMs. Additionally, oral delivery of the liquid formulation provides a platform for feasibility and compliance, given the preference for oral dosing by patients, especially for chronic administration and for the administration of a therapeutic outside of hospital settings. HBI-002, a liquid CO drug product developed by Hillhurst Biopharmaceuticals, has been shown to be effective, including reducing inflammation, in animal models of SCD [89], Parkinson’s disease [90] and anthracycline-induced cardiotoxicity [91], among other areas. HBI-002 is currently undergoing clinical testing. A Phase 1 clinical trial (NCT03926819) to assess the safety and pharmacokinetics of HBI-002 in healthy volunteers was completed and showed no adverse events of clinical significance. In addition, a Phase 2a clinical trial [NCT 06144749] in subjects with SCD is currently enrolling, and a Phase 2a clinical trial [NCT07005180] in subjects with Parkinson’s disease is reported to begin soon.

We also present a comparative overview of inhaled CO, CORMs, organic prodrugs, and oral CO formulations, as summarized in Table 4, highlighting significant differences among current and emerging CO delivery modalities. These delivery systems vary widely in biodistribution, safety profile, COHb impact, and translational readiness.

Systemic COHb levels may not accurately reflect retinal availability because of hemoglobin buffering and limitations of the blood-retinal barrier. Safe systemic doses may be subtherapeutic for focal diseases such as retinal vein occlusion, whereas localized strategies for these conditions can focus CO at the target with low off-target effects. Future investigations should focus on comparing systemic versus intravitreal, implant, or suprachoroidal routes with similar structural, molecular, and functional endpoints. Direct or surrogate intraocular readouts, in addition to systemic COHb, could be included in future studies. Ocular PBPK (Physiologically Based Pharmacokinetic) may be used to guide dose selection. Optimizing strategies could come from new paradigms—intravitreal CORM nanoparticles and biodegradable depots (to achieve sustained release of this molecule); refillable, port-like reservoirs (to reduce procedure burden); enzyme- or trigger-activated CORMs (to bias release within retinal tissues); suprachoroidal depots (to cover the posterior side region); and liquid CO formulations (oral or intravitreal-ready) to yield accurate titration, rapid activation, adaptive compounding, simplicity of manufacture and scaling, and simple route adaptation. Although evidence is still being collected, these approaches may clearly resolve concerns regarding retinal exposure, disease-applicable targeting, and the systemic dose ceiling, providing a realistic and optimistic approach to CO translation in the eye.

## 7. Safety Considerations

### 7.1. Toxicity and Dosage

Studies have shown that COHb levels below 10% are generally well tolerated in humans, with minimal side effects such as headaches or discomfort. For inhaled CO, safe dosage ranges typically fall between 100 and 250 ppm, depending on the duration and frequency of exposure [92]. Clinical trials in patients with COPD, idiopathic pulmonary fibrosis, and sepsis-induced ARDS, among other areas, have used these ranges with good tolerability. Monitoring COHb levels is essential to avoid toxicity. Blood gas machines with co-oximetry that directly measure COHb from venous blood samples are widely used and readily available, and, for inhaled CO, algorithms like the Coburn-Forster-Kane (CFK) equation have proven effective in indirectly predicting safe COHb levels with inhaled CO [93]. Side effects are rare at therapeutic doses but may include mild hypoxia, especially in individuals with pre-existing respiratory conditions. The development of CORMs and oral liquid CO drug products targets improved safety profiles by carefully controlling CO dosing, minimizing systemic COHb elevation, and/or enhancing tissue specificity.

### 7.2. Regulatory and Ethical Issues

The regulatory landscape for CO therapy is evolving. CO is classified as a medical gas by the FDA and other regulatory agencies [94], and is used widely in pulmonary function testing (diffusion capacity of the lungs). At the same time, the potential toxicity of CO at high exposure is well known. The clinical testing of CO has taken place under the auspices of various regulatory agencies, including the FDA, EMA, and others. Numerous Phase I and II trials have been allowed by these regulatory agencies to assess the safety and, in certain studies, efficacy of inhaled CO, CORMs, and oral liquid CO [73]. As with the development of all drugs, ethical considerations center around the balance between therapeutic benefit and potential toxicity. Informed consent, rigorous safety monitoring, and transparent communication of risks are essential in clinical trials, as is careful ethical oversight. Additionally, as is required in drug development, the potential for off-target effects and long-term consequences of the use of CO must be addressed through the appropriate preclinical and clinical evaluations.

## 8. Prospects and Future Directions

### 8.1. Potential for Broader Applications

CO therapy, initially explored for retinal ischemia-reperfusion injury, holds promise for a broader spectrum of ocular diseases. In glaucoma models, CO has demonstrated the ability to lower intraocular pressure and protect retinal ganglion cells from apoptosis and inflammation. Autoimmune uveitis models have benefited from CO’s immunomodulatory effects, including suppression of Th17 responses and enhancement of IL-10 expression. Beyond the eye, CO therapy has shown therapeutic potential in systemic cardiovascular, pulmonary, hepatic, and renal diseases, mainly due to its anti-inflammatory, anti-apoptotic, and vasodilatory properties. These findings suggest that CO-based treatments could be extended to other neurodegenerative and inflammatory conditions, provided delivery methods and safety profiles are optimized for each application.

### 8.2. Research Gaps and Challenges

Despite encouraging preclinical and early clinical data, several challenges must be addressed before CO therapy can be widely adopted. Mechanistically, the precise molecular pathways through which CO exerts its protective effects in ocular tissues remain incompletely understood, particularly regarding its interactions with MAPK signaling and HO-1 regulation. Delivery methods also pose significant hurdles; inhaled CO lacks tissue specificity, poses dosing issues, and carries risks of systemic toxicity, while CORMs require further refinement in pharmacokinetics, stability, and safety. Oral liquid drug products appear promising, but have yet to be assessed in large, later stage clinicals trials. Clinical trials have demonstrated appropriate safety but limited efficacy, potentially due to subtherapeutic dosing or poor tissue targeting. Regulatory approval is contingent on demonstrating appropriate benefit:risk, along with clinical feasibility. Future research should prioritize mechanistic studies, delivery optimization, and translational trials to fully realize the therapeutic potential of CO in retinal and systemic diseases.

## 9. Conclusions

The discovery of endogenous CO synthesis via HO-1 has positioned both HO-1 activators and CO as promising therapeutic agents. Both have demonstrated beneficial effects in preclinical models of various diseases, including ocular pathologies such as retinopathy and age-related degeneration. However, the precise molecular mechanisms by which HO-1 and CO exert their protective effects remain incompletely understood. Future research should aim to delineate these pathways more clearly, particularly focusing on the modulation of oxidative stress, inflammation, and apoptosis.

For HO-1, the development of more selective inducers is critical to minimize off-target effects and enhance therapeutic precision. Comparative studies evaluating the efficacy of HO-1 induction versus direct CO administration are also needed to guide clinical translation. Although human studies involving CO inhalation have shown safety, they have largely failed to yield significant therapeutic outcomes-possibly due to subtherapeutic dosing. Future trials should consider higher inhaled CO doses with rigorous monitoring of vital signs and COHb levels to ensure both safety and efficacy.

CORMs offer a delivery strategy that has the potential to circumvent systemic CO exposure and COHb elevation. These compounds can be designed to localize CO release to target tissues, making them attractive candidates for ocular therapy. Comprehensive studies on the pharmacokinetics, pharmacodynamics, and toxicity profiles of CORMs are essential to determine their suitability for human use. Additionally, nanomaterial-based delivery systems may enhance CORM stability and tissue specificity, particularly in the context of retinal disease.

Importantly, HBI-002, an orally bioavailable CO drug product, represents a novel and practical alternative to both inhaled CO and CORMs. Its ease of administration as compared with inhaled CO, favorable safety profile as compared with CORMS, and supportive clinical data thus far make it a compelling candidate for further investigation in ocular disease models. Future research should explore its therapeutic potential, optimal dosing strategies, and long-term effects in retinal pathologies.

In summary, HO-1 activation and CO, whether delivered via inhalation, CORMs, or drug products like HBI-002, hold significant promise as therapeutic agents for retinal diseases. Continued research is essential to refine and translate these approaches into safe, effective treatments for patients suffering from vision-threatening conditions.

## Figures and Tables

**Table 1:** Therapeutic use of Carbon monoxide in preclinical models of retinal diseases.

Methods of Administration	Retinal Disease Models	Dose & Timing of Administration	Effects	References
Inhaled CO gas	Rat retinal ischemia/reperfusion (I/R) injury model	Preconditioning: 250 ppm CO inhalation for 1 h prior to induction of ischemia	Neuroprotection: Reduction in RGC death vs. controls 7 days after I/R injury. Anti-apoptotic: Attenuated caspase-3 activity and reduced TNF-α protein expression. Stress response: Increased HSP-70 protein expression in retina after CO pre-conditioning. Transcription factor modulation: Regulation of CREB and HSF-1; suppression of I/R-induced NF-κB DNA. MAPK signaling: CO influenced phosphorylation of MAPKs (e.g., effects on pERK1/2, p-p38).	[57]
Inhaled CO gas	Rat retinal I/R injury model.	Postconditioning: 250 ppm CO inhalation for 1 h at reperfusion or delayed to 1.5 h or 3 h after reperfusion to assess therapeutic window.	Neuroprotection: Significant increase in RGC survival at 7 days in CO-treated groups vs. I/R alone across immediate, 1.5 h, and 3 h post-treatment windows. Anti-apoptotic: Reduced expression of Bax and caspase-3 (pro-apoptotic factors) and increased Bcl-2 (anti-apoptotic) mRNA/protein levels. Anti-inflammatory: Inhibition of NF-κB activation and reduced microglial and Müller glia activation and decreased immigration of proliferating microglia/macrophages into the retina. MAPK signaling modulation: CO postconditioning decreased p38 MAPK phosphorylation and increased ERK-1/2 MAPK phosphorylation in retinal tissue; these shifts are associated with anti-apoptotic and cell survival signaling. Oxidative stress/HO-1: CO reduced oxidative stress markers and modulated HO-1 expression in the retina.	[58]
Inhaled CO gas	Rat retinal I/R injury model.	Postconditioning: 250 ppm CO inhalation for 1 h immediately at reperfusion.	Neuroprotection: Increased RGC survival following I/R injury compared with untreated I/R controls. Anti-inflammatory: Marked reduction in microglial activation and decreased number of activated microglia/macrophages in the ischemic retina. Cytokine modulation: Reduced expression of pro-inflammatory mediators including TNF-α and IL-1β. NF-κB signaling: Suppression of NF-κB activation in retinal tissue after I/R injury. Glial response modulation: Attenuation of reactive gliosis in the ischemic retina.	[55]
Inhaled CO gas	Rat optic nerve crush (ONC) model	Postconditioning: 250 ppm CO inhalation for 1 h following optic nerve crush.	Neuroprotection: Increased survival of RGCs after optic nerve crush compared with untreated controls. Anti-apoptotic: Reduced apoptotic cell death in retinal tissue (decreased cleaved caspase-3) and modulation of Bcl-2/Bax expression favoring cell survival. MAPK signaling modulation: Increased ERK1/2 phosphorylation and decreased p38 MAPK phosphorylation, consistent with pro-survival signaling. Mitochondrial protection: Attenuation of mitochondrial-mediated apoptotic pathways in injured retinal neurons.	[49]
Inhaled CO gas	Rat ONC model	Preconditioning: 250 ppm CO inhalation for 1 h administered prior to optic nerve crush	Neuroprotection: Significant increase in RGC survival following optic nerve crush compared with untreated ONC controls. Anti-apoptotic: Decreased expression of cleaved caspase-3 and Bax; increased Bcl-2 expression, indicating suppression of the mitochondrial apoptotic pathway. Apoptotic pathway inhibition: Reduced activation of downstream apoptotic signaling following axonal injury. Cell survival signaling modulation: Regulation of MAPK signaling pathways consistent with enhanced pro-survival signaling.	[50]
Intravenous (IV) Injection of CORM (ALF-186)	Rat retinal I/R injury model.	Postconditioning: IV injection 10 mg/kg ALF-186 administered immediately after induction of I/R.	Neuroprotection: Increased RGC survival following I/R injury compared with untreated controls. Anti-apoptotic: Reduced cleaved caspase-3 expression and decreased apoptotic cell death in retinal tissue. MAPK signaling modulation: Activation (phosphorylation) of p38 MAPK associated with anti-apoptotic signaling in this context; modulation of downstream survival pathways. Apoptotic pathway regulation: Inhibition of mitochondrial-dependent apoptotic signaling mechanisms following ischemic injury.	[59]
IV of CORM (ALF-186)	Rat retinal I/R model injury.	Postconditioning: ALF-186 was administered at 10 mg/kg body weight via a single i.v. injection immediately after induction of retinal ischemia. The soluble guanylate cyclase (sGC) inhibitor ODQ was administered prior to ALF-186 to assess sGC-dependence of the neuroprotective effects.	Neuroprotection: Increased retinal ganglion cell (RGC) survival following I/R injury compared with untreated I/R controls. Anti-inflammatory: Reduced microglial activation and decreased expression of pro-inflammatory cytokines (including TNF-α and IL-1β). sGC signaling activation: Neuroprotective and anti-inflammatory effects mediated via activation of sGC β1 and downstream cGMP signaling pathways. Anti-apoptotic: Decreased apoptotic cell death in retinal tissue with reduced cleaved caspase-3 expression. Signal pathway modulation: Involvement of cGMP-dependent mechanisms linking CO release to suppression of inflammatory and apoptotic signaling after ischemic injury.	[54]
Intravitreal (i.v.) injection of CORM (ALF-186)	Rat ONC model	Postconditioning: i.v. of ALF-186 (1 mM solution, 5 µL) administered immediately after ONC.	Neuroprotection: Increased RGC survival following optic nerve crush compared with untreated ONC controls. Neuroregeneration: Enhanced axonal regeneration distal to the crush site, indicating promotion of regenerative capacity beyond simple survival. Anti-apoptotic: Reduced apoptotic signaling within retinal ganglion cells following injury. Axonal growth signaling modulation: Activation of intracellular pathways associated with neuronal regeneration and survival.	[56]
Topical Ocular administration of CORM-3 (topical)	Normotensive rabbit model assessing intraocular pressure (IOP); not an injury model.	Topical: instillation of 50 µL of a 100 µM CORM-3 solution applied to the eye; IOP measured at multiple time points following administration. 0.001–1% after induction	IOP reduction: Significant decrease in intraocular pressure following topical CORM-3 administration compared with baseline and vehicle-treated controls. sGC involvement: IOP-lowering effect associated with activation of the sGC/cGMP pathway. Mechanistic implication: Findings suggest CO-mediated modulation of aqueous humor dynamics via cGMP-dependent signaling.	[51]
Intraperitoneal(i.p.) injection of CORM-A1	Rat experimental autoimmune uveoretinitis (EAU) model.	Post induction therapeutic treatment: CORM-A1 was administered via i.p. at 5 mg/kg once daily from day 9 to day 14 after EAU induction.	Clinical improvement: Significant reduction in clinical EAU severity scores compared with untreated EAU controls. Anti-inflammatory: Decreased retinal inflammatory cell infiltration and reduced expression of pro-inflammatory cytokines (including TNF-α and IFN-γ). Immunomodulatory effects: Suppression of Th1/Th17-associated immune responses implicated in autoimmune retinal injury. Neuroprotective implication: Reduced structural retinal damage associated with autoimmune inflammation.	[53]

CO, carbon monoxide; I/R, ischemia/reperfusion; ppm, parts per million; RGC, retinal ganglion cell; TNF-α, tumor necrosis factor alpha; HSP-70, heat shock protein 70; CREB, cAMP response element-binding protein; HSF-1, heat shock factor-1; NF-κB, nuclear factor kappa-light-chain-enhancer of activated B cells; MAPK, mitogen-activated protein kinase; MAPKs, mitogen-activated protein kinases; ERK1/2, extracellular signal-regulated kinase 1/2; pERK1/2, phosphorylated extracellular signal-regulated kinase 1/2; p38 MAPK, p38 mitogen-activated protein kinase; p-p38, phosphorylated p38 MAPK; Bax, B-cell lymphoma-2-associated X protein; Bcl-2, B-cell lymphoma-2; HO-1, heme oxygenase-1; ONC, optic nerve crush; CORM, carbon monoxide-releasing molecule; ALF-186, a water-soluble carbon monoxide-releasing molecule; IV, intravenous; i.v., intravitreal; sGC, soluble guanylate cyclase; ODQ, 1H-[1,2,4]oxadiazolo[4,3-a]quinoxalin-1-one (sGC inhibitor); cGMP, cyclic guanosine monophosphate; IL-1β, interleukin-1 beta; IOP, intraocular pressure; EAU, experimental autoimmune uveoretinitis; IFN-γ, interferon gamma; Th1, T helper type 1 cells; Th17, T helper type 17 cells; i.p., intraperitoneal; µL, microliter; mM, millimolar; Müller glia, retinal Müller glial cells.

**Table 2:** Selected clinical trials with carbon monoxide therapy to treat non-ocular diseases.

Method of Administration	Disease/Population	Dose & Timing	Effects/Notes	Status	ClinicalTrials.Gov ID
Inhaled CO	Stable COPD	100–125 ppm for 2 h × 4 consecutive days	Reduction in sputum eosinophils and improved bronchial responsiveness	Completed (2006)	NCT00122694
Inhaled CO	Pulmonary inflammatory response after endotoxin instillation/ARDS model in healthy volunteers	CO via mask for 6 h	No significant anti-inflammatory effect; pilot and main studies both completed	Completed (2010)	NCT00094406
Inhaled CO	Idiopathic Pulmonary Fibrosis (IPF)	100–200 ppm, 2 h per dose, twice weekly × 12 weeks	No significant differences in physiologic measures, acute exacerbations, hospitalization, death, or PROs	Completed (2017)	NCT01214187
Inhaled CO	Sepsis-induced ARDS	100 or 200 ppm for 90 min up to 5 days	CFK equation highly accurate for predicting COHb	Completed (2019)	NCT02425579
Inhaled CO	Sepsis-induced ARDS (personalized CFK-based dosing)	200–500 ppm for 90 min daily × 3 days to target COHb 6–8%	Safety and CFK accuracy under evaluation	Active, not recruiting	NCT04870125
Inhaled CO	ARDS in mechanically ventilated patients	200 ppm for 90 min daily × 3 days	Evaluating safety, tolerability, and biologic readouts	Active, not recruiting	NCT03799874
Liquid CO (HBI-002)	Healthy adult volunteers	Single ascending dose + multiple daily doses × 7 days	Phase 1 safety and PK study of oral CO formulation	Completed	NCT03926819

CO, carbon monoxide; ppm, parts per million; COPD, chronic obstructive pulmonary disease; ARDS, acute respiratory distress syndrome; IPF, idiopathic pulmonary fibrosis; PROs, patient-reported outcomes; CFK, Coburn–Forster–Kane equation; COHb, carboxyhemoglobin; PK, pharmacokinetics; HBI-002, oral liquid carbon monoxide formulation; NCT, National Clinical Trial identifier (ClinicalTrials.gov); min, minutes; h, hours; ×, times; %, percent.

**Table 3:** Evidence hierarchy map for carbon monoxide (CO)–based therapeutic effects in ocular disease models.

Indication	Model/Data Type	Observed Effect of CO Modulation	Human Evidence?	Main Limitation
Retinal Ischemia–Reperfusion Injury	Rodent models, cellular oxidative-stress assays	Reduced apoptosis, decreased inflammatory markers, improved retinal function	No direct trials	Preclinical models may not fully mirror human ischemic timing and severity
Autoimmune Uveitis	Mouse EAU model	Suppressed inflammatory cytokines, reduced immune-cell infiltration	No	Autoimmune mechanisms in EAU differ from human uveitis heterogeneity
Ocular Hypertension/Glaucoma-Related Injury	Rodent ocular-hypertension models	Neuroprotection of retinal ganglion cells, reduced oxidative stress	No	Lack of long-term pressure-modulation studies; no clinical trials
General Ocular Anti-inflammatory and Cytoprotective Effects	Multiple preclinical models	HO-1 induction decreases oxidative stress and inflammation	Indirect support from systemic early-phase CO trials	Existing human CO trials assess safety/tolerability but not ocular endpoints
Systemic CO Therapy (safety/tolerability)	Phase I/II human trials (non-ocular indications)	Demonstrated dose-dependent tolerability with controlled administration	Yes	No ocular efficacy data; mixed systemic efficacy in unrelated diseases

CO, carbon monoxide; EAU, experimental autoimmune uveitis; HO-1, heme oxygenase-1; Phase I/II, phase one/phase two clinical trials.

**Table 4:** Comparison of CO Delivery Methods.

Delivery Method	Tissue Targeting	Impact on Carboxyhemoglobin (COHb)	Development Stage	Known Adverse Events
Inhaled CO	Systemic, non-selective; CO distributes widely due to inhalation physiology	Can elevate COHb depending on dose; monitored closely in trials	Early-phase human trials for non-ocular indications showing tolerability	Headache, dizziness, risk of CO toxicity at high exposures; requires controlled inhalation systems
CORMs (Carbon Monoxide-Releasing Molecules)	Potential for targeted release depending on structure and trigger (e.g., metal-based, enzymatic, photolabile)	Minimal COHb effect because CO is released intracellularly or in micro-environments	Preclinical (cell and animal studies); numerous CORM families under development	Metal residues from some CORMs can contribute to toxicity; variable CO-release kinetics
Organic CO Prodrugs	Designed for controlled CO release in specific tissues; improved selectivity over direct inhalation	Lower systemic COHb levels expected vs. inhalation due to localized release	Preclinical; emerging drug-design platforms reported	Limited by formulation stability; dose-dependent GI tolerance in some models
Oral CO-Containing Liquids/Enteral CO	Enteral absorption allows gradual systemic distribution; potential for targeting GI-associated or hepatic tissues	COHb increases are modest and slower vs. inhalation due to enteral absorption kinetics	Patented formulations; early translational stage (preclinical ± regulatory filings)	GI discomfort, variable CO release depending on formulation

CO, carbon monoxide; COHb, carboxyhemoglobin; CORMs, carbon monoxide-releasing molecules; GI, gastrointestinal.

## Data Availability

No new data were created or analyzed in this study.

## Abbreviations

The following abbreviations are used in this manuscript:

Akt	Protein kinase B
AIF-1	Allograft inflammatory factor 1
AMPK	AMP-activated protein kinase
CAT	Catalase
CO	Carbon monoxide
COHb	Carboxyhemoglobin
CoPP	Cobalt protoporphyrin
CORM	Carbon monoxide-releasing molecule
CREB	cAMP response element-binding protein
ERK1/2	extracellular-signal-regulated kinase-1/2
GPx	Glutathione peroxidase
GSH	Glutathione
GTR	Glutathione reductase
Hb	Hemoglobin
HO-1	Heme oxygenase 1
HSP70	Heat shock protein 70
IL-1b	Interleukin-1-beta
IL-6	Interleukin-6
IL-10	Interleukin-10
IL-17	Interleukin-17
IOP	Intraocular pressure
IRI	Ischemia-reperfusion injury
iNOS	inducible nitric oxide synthase
JNK1/2	c-Jun N-terminal kinase-1/2
Keap1	Kelch-like ECH-associated protein-1
LPS	Lipopolysaccharide
NO	Nitric oxide
O_2_	Oxygen
ONC	Optic nerve crush
MAPK	Mitogen-activated protein kinase
MCP-1	monocyte chemoattractant protein-1
MDA	Malondialdehyde
mTORC1	Akt/mammalian target of rapamycin complex 1
NF-kB	nuclear factor kappa-light chain-enhancer of activated B cells
Nrf2	Nuclear factor erythroid 2-related factor-2
PI3K	phosphatidylinositol 3-kinase
RGC	Retinal ganglion cell
ROS	Reactive oxygen species
RPE	Retinal pigment epithelial cells
Sal A	Salvianolic acid A
sGC	Soluble guanylate cyclase
SCD	Sickle cell disease
SOD	superoxide dismutase
StRE	Stress response element
Treg Cells	Regulatory T cells
Th17 Cells	T helper 17 cells
TNF-α	Tumor necrosis factor alpha
VEGF	Vascular endothelial growth factor
